# Role of WT1 in Measurable Residual Disease Follow-Up in the Post Allogeneic Stem Cell Transplant Setting

**DOI:** 10.3390/jcm13175145

**Published:** 2024-08-30

**Authors:** Sinem Namdaroğlu, Semih Başcı, Burcu Aslan Candır, Samet Yaman, Tuğçe Nur Yiğenoğlu, Taha Bahsi, Nurgül Özcan, Mehmet Sinan Dal, Merih Kızıl Çakar, Fevzi Altuntaş

**Affiliations:** 1 Department of Hematology and Bone Marrow Transplantation Center, Dokuz Eylul University Hospital, Izmir 35330, Turkey; semih.basci@gmail.com; 2 Department of Hematology and Bone Marrow Transplantation Center, Ankara Dr. Abdurrahman Yurtaslan Oncology Training and Research Hospital, University of Health Sciences, Ankara 06200, Turkey; bcandir@yahoo.com (B.A.C.); syaman@yahoo.com (S.Y.); tnur@gmail.com (T.N.Y.); sdal@yahoo.com (M.S.D.); mcakar@gmail.com (M.K.Ç.); faltunt@yahoo.com (F.A.); 3 Department of Genetics, Ankara Dr. Abdurrahman Yurtaslan Oncology Training and Research Hospital, University of Health Sciences, Ankara 06200, Turkey; tbasi@gmail.com

**Keywords:** allogeneic hematopoietic cell transplantation, acute myeloid leukemia, Wilms’ tumor 1, chimerism, flow cytometry, polymerase chain reaction

## Abstract

**Objectives**: The Wilms’ tumor gene 1 (WT1) plays a critical role in cell development and the regulation of essential genes involved in cell growth and metabolism. In the context of hematopoietic tumors, including acute myeloid leukemia (AML), WT1 has been identified as a potential marker for measurable residual disease (MRD) assessment. Relapse after allogeneic hematopoietic stem cell transplantation (allo-SCT) remains a significant challenge in AML treatment, highlighting the importance of MRD monitoring for risk stratification and treatment decisions. This study aimed to investigate the clinical significance of WT1 as a molecular marker for MRD and its correlation with chimerism in AML patients post-allo-SCT setting. **Methods:** We have included 58 patients with WT1-expression-positive acute myeloid leukemia (AML) who received allo-SCT in our center between 2016–2022. The exclusion criteria are as follows: not having WT1 polymerase chain reaction (PCR) measurement at diagnosis, not receiving allo-SCT, and not having a serial measurement of WT1 post-transplant. Pre- and post-transplant assessments were made with flow cytometry, WT1 PCR, and bone marrow morphological evaluations. Statistical analyses were carried out to explore correlations between WT1 levels, MRD markers, and chimerism post-transplantation. **Results:** We found that WT1 had a significant correlation with flow cytometry and bone marrow morphological evaluation, but not with chimerism. Interestingly, high WT1 expressors exhibited a more robust correlation with chimerism compared to the general cohort. The negative predictive value for post-allo-SCT relapse was 91.8% for the whole WT1 cohort; for high WT1 expressors, it was similar, at 87.5%. The negative predictive value for post-allo-SCT relapse was high for the whole WT1 cohort; for high WT1 expressors, it was similar. The WT1 MRD assay showed a high negative predictive value for post-allo-SCT relapse, consistent across both the entire cohort (91.8%) and high WT1 expressors (87.5%). **Conclusions:** WT1 expression levels may serve as a valuable ancillary marker in MRD assessment and relapse prediction post-allo-SCT in AML patients, particularly for those lacking specific fusion genes or mutations. However, further large-scale, controlled studies are needed to standardize WT1 MRD assays and establish clear guidelines for their clinical application.

## 1. Introduction

The Wilms’ tumor gene 1 (WT1) was first discovered as a tumor suppressor gene implicated in the etiology of pediatric renal Wilms’ tumor and is located on chromosome 11p13q [1,2]. WT1 plays a crucial role in cell development and functions as an active transcription factor due to the presence of zinc fingers in its C-terminal region. These zinc fingers contribute to WT1’s role in regulating essential genes involved in cell growth and metabolism [3].

WT1 exhibits dual roles in the regulation of target genes, acting either as a promoter or a suppressor, depending on factors such as WT1 expression levels, isoforms, transcription site location, and cell type [4,5]. Its significant presence is observed in various hematopoietic tumors, including acute myeloid leukemia (AML), as well as in healthy marrow cells [6,7]. In hematopoietic cells, WT1 seems to function as a tumor suppressor gene. The overexpression of WT1 in young bone marrow (BM) cells leads to cell proliferation arrest and restricted colony development. In healthy individuals, WT1 is expressed at exceptionally low quantities and is limited to the early CD34+ cell population [8,9].

Relapse stands out as a primary contributor to therapeutic failures in individuals with AML after allogeneic hematopoietic stem cell transplantation (allo-SCT), frequently manifesting as a refractory condition. Recent evidence indicates that measurable residual disease (MRD) serves as a distinctive and prognostic indicator for AML. The assessment of MRD is crucial for risk stratification and treatment decisions alongside conventional pre- treatment clinical, cytogenetic, and molecular data [10,11,12].

In 2017, the European Leukemia Net (ELN) introduced a novel response criterion for MRD-negative complete remission (CR) [13]. According to ELN guidelines, real-time quantitative PCR (RT-PCR) is recommended as the gold standard for AML cases exhibiting specific abnormalities like PML-RARA, RUNX1-RUNX1T1, CBFB-MYH11, or NPM1, encompassing 40% of AML patients [10].

Flow cytometry (FC) has served as the gold standard for determining MRD in patients with acute lymphoblastic leukemia. However, around 20% of AML patients lack the necessary markers for MRD follow-up, and changes in the initial immunophenotype can occur during relapse after allo-SCT [14]. In contrast, a significant subset of AML patients does not exhibit specific fusion genes or mutations. Pan-leukemic markers such as WT1, which is commonly overexpressed in AML, represent potential targets for molecular MRD screening [15,16,17].

The use of the molecular screening of WT1 for predicting and managing relapse after allo-SCT has been considered questionable [18]. Researchers associated with ELN have introduced an enhanced and standardized WT1 test, establishing reference thresholds for both peripheral blood and BM [19]. However, the achieved cut-off values have been inconsistent, and, as of now, there is no consensus, limiting their application in clinical practices [17].

While several studies suggest the potential role of WT1 in MRD assessment during chemotherapy and allo-SCT, the WT1 MRD assay has not garnered sufficient attention and is not commonly utilized in AML due to the absence of large-scale, controlled studies [12]. Moreover, there are no clear recommendations regarding optimal timeframes, reference ranges, or candidates for the WT1 MRD assay in comparison to FC, and a correlation with chimerism has been suggested [12,14,20,21].

Our objective here is to explore the clinical significance of WT1 in MRD and chimerism in the post-allo-SCT setting.

## 2. Methods

### 2.1. Patients and Data

We retrospectively included 58 patients with AML who had allo-SCT at our facility between 2016 and 2022. Exclusion criteria comprised individuals without WT1 PCR measurements at the time of diagnosis, those who did not undergo allo-SCT, and those lacking serial measurements of WT1 post-transplant.

This study was conducted in accordance with the ethical principles outlined in the Declaration of Helsinki and received approval from the local Institutional Review Board. Patient demographics, AML-related data (such as white blood count at diagnosis, cytogenetic results at diagnosis, and European Leukemia Network risk status), and treatment details are presented in Table 1. Additional information on allo-SCT features is provided in Table 2.

### 2.2. Laboratory Evaluations

Measurable residual disease (MRD) assessment for patients was utilized using flow cytometry, WT1 RT-PCR, and bone marrow evaluation (morphological assessment of blast number) sequentially at specific time points including diagnosis, post-induction, pre- and post-transplant period.

#### 2.2.1. Flow Cytometry

Flow cytometric analyses of samples were performed with Becton Dickinson, San Jose, CA, USA (BD). The cells were harvested using the whole blood lyse-washing technique within 2–4 h after sampling, following the sample preparation protocol provided by the manufacturer.

#### 2.2.2. WT1 RT-PCR

Quantitative assessment of the WT1 transcript amount and other AML-related mutations was conducted using quantitative RT-PCR. To assess WT1 expression, we used the ELN Ipsogen^®^ WT1 ProfileQuant^®^ Kit (CE-approved kit, Ref 676923, QIAGEN GmbH, Marseille, France) to perform a quantitative polymerase chain reaction (RT-PCR) test on bone marrow samples at related time points, which was designed on exons 1 and 2 of WT1, in accordance with the European Leukemia Net protocol.

#### 2.2.3. Chimerism Analysis

Chimerism analyses were conducted using the short tandem repeat PCR (STR-PCR) method. Chimerism was routinely assessed post-transplant, with monthly evaluations for the first three months and subsequent assessments every three months. In clinically indicated situations, such as mixed chimerism, chimerism failure, or relapsed disease, more frequent assessments were performed on a patient basis.

#### 2.2.4. Bone Marrow Evaluation (Morphological Assessment of Blast Count)

The bone marrow evaluation was carried out pre-transplant, post-transplant first month, post-transplant third month, and when suspicion of relapse was raised.

### 2.3. Statistical Analyses

Data processing was conducted using IBM SPSS Statistics for Windows (Version 26.0, Armonk, NY, USA) software. Descriptive statistics were employed, with categorical variables reported as numbers and percentages, while quantitative variables were presented as medians (minimum–maximum). Normal distribution of variables was assessed using the Kolmogorov–Smirnov test. Differences among categorical variables were examined using the χ^2^ test, with further comparisons made when necessary. Correlation analyses were performed using the Spearman correlation test. A two-sided *p*-value of ≤0.05 was considered statistically significant.

## 3. Results

The cohort included 58 patients, with 34 (58.6%) males and 24 (41.4%) females. AML NOS was the most prevalent type, observed in 41 patients (70.7%). FLT3 was the most frequently encountered genetic abnormality, identified in 21 cases (36.2%), and isolated WT1 tested positive in 17 cases (29.3%). Among the cases, 34 (58.6%) were classified in the adverse risk cytogenetic group, while 23 (39.7%) fell into the intermediate cytogenetic risk group. The majority of patients (50–86.2%) achieved CR before transplantation. Busulfan-based conditioning was the most commonly employed regimen. Patient characteristics and transplant-related data are presented in Table 1. WT1 expression was classified as intermediate to high in 26 patients (44.8%), with 500 copies or more, and as low in 32 patients (55.2%), with less than 500 copies. The sequential assessment of WT1 measurements, flow cytometry, bone marrow evaluation, and chimerism results is presented in Table 2. Correlations between WT1 levels, flow cytometry results, bone marrow evaluations, and chimerism at specific time points were examined. A statistically significant association was found between WT1 levels and flow cytometry, as well as between WT1 levels and bone marrow evaluation. Additionally, a strong correlation was observed between flow cytometry and bone marrow evaluation, both of which were associated with WT1. However, no significant correlation was found between chimerism and WT1 in the post-transplantation setting.

**Figure 1:**
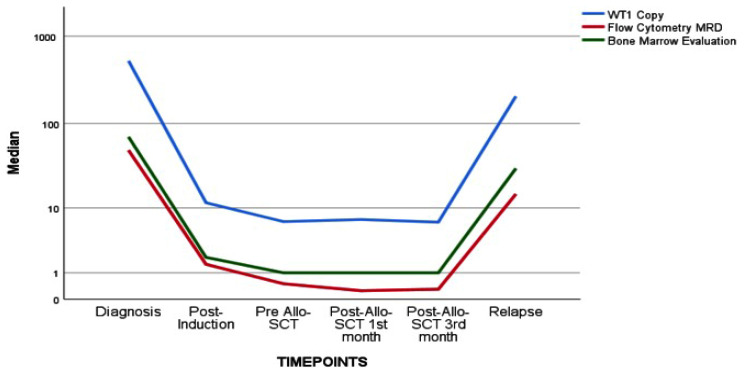
The relationship of WT1 assay and flow cytometry and bone marrow evaluation.

In a more detailed analysis, we conducted a separate examination of high WT1 expressors and their correlation with MFC, BME, and chimerism. The correlation coefficient remained similar for the entire WT1 cohort and high WT1 expressors in both MFC and BME. Interestingly, the association between chimerism and high WT1 expressors was much more robust in the subgroup analysis. The negative predictive value for post-allo-SCT relapse was further analyzed with pre-allo WT1 MRD results, revealing a value of 91.8% for the entire WT1 cohort and a similar 87.5% for high WT1 expressors. The correlation is illustrated in Table 3, and the graphical representation of WT1, flow cytometry, and bone marrow evaluation is presented in Figure 1.

## 4. Discussion

In our study, we observed a significant correlation between WT1 and flow cytometry as well as bone marrow evaluation, although no correlation was found with chimerism. Interestingly, high WT1 expressors demonstrated a significant correlation with chimerism. The negative predictive value for post-allo-SCT relapse was high for the entire WT1 cohort, and it remained similar for high WT1 expressors. Further analysis of the negative predictive value for post-allo-SCT relapse with pre-allo WT1 MRD results revealed values of 91.8% for the whole WT1 cohort and a similar 87.5% for high WT1 expressors. These findings suggest that WT1 can serve as an ancillary marker, aiding clinicians in decision-making. WT1 is notably expressed in various hematopoietic tumors, including AML, and is a key molecule influencing cell development. In AML patients undergoing allo-SCT, relapse poses a significant challenge to treatment efficacy and often manifests as a refractory condition. The emerging understanding underscores MRD as a distinctive and prognostic predictor for AML, playing a crucial role in risk stratification and treatment decisions alongside traditional pre-treatment clinical, cytogenetic, and molecular data. While the current gold standard for MRD assessment involves PCR targeting specific markers, applicable to only 40% of AML cases, around 20% of patients lack these markers for effective MRD follow-up. Moreover, shifts in the initial immunophenotype during relapse post-allo-SCT further complicate monitoring efforts [15].

On the contrary, a significant subset of AML patients lacks distinctive fusion genes or mutations. Pan-leukemic markers like WT1, typically exhibiting an overexpression in AML, emerge as promising targets for molecular MRD screening [15,16,17]. Establishing an accurate reference serves as the foundation for defining specific cut-off values of WT1, crucial for predicting relapse. ELN researchers have proposed BM WT1 MRD positivity at more than 250 copies as a threshold to distinguish it from baseline increases [20]. In a prospective study, Byung Sik Cho et al. included 425 patients and assessed BM WT1 MRD pre-allo-SCT and post-allo-SCT at 1 or 3 months. Their findings highlighted the utility of the WT1 MRD assay across all AML types, with the post-allo third-month evaluation proving to be particularly instructive for relapse prediction. Similarly, Rossi et al. demonstrated the predictive role of post-transplant WT1 evaluation through both univariate and multivariate analyses at 1 month after allo-SCT [21]. Lambert et al. found that patients with MRDhigh (WT1 MRD ratio > 2.5% in BM or > 0.5% in PB) after induction had a higher risk of relapse and a shorter RFS and OS. The early WT1 MRD response highlights a population of high-risk patients in need of additional therapy [22].

Recently, Qin et al. validated that a WT1 level exceeding 0.6% post-allo-SCT predicts relapse, consistent with earlier findings. They further demonstrated that, in specific fusion transcript t(8;21)-positive AML, the combined evaluation of the WT1 MRD assay with a threshold of WT1 > 1.8% proved to be a more accurate predictor for relapse [17].

Malagola et al. included 24 patients and analyzed WT1 RT-PCR from peripheral blood (PB) and BM before and after allo-SCT. They showed five copies of WT1/ABL × 104 from PB as the threshold value that correlated with relapse after allo-SCT but not correlated with BM WT1 RT-PCR [23]. Rautenberg et al. evaluated 64 AML/MDS patients for WT1 levels in their peripheral blood prior to allo-SCT [24]. The two-year post-transplant CIR, RFS, and OS were similar in WT1-MRD-positive and refractory patients at transplant (61% vs. 70%, 37% vs. 26%, and 54% vs. 56%, respectively), but much better in WT1-MRD-negative cases (10%, 89%, and 90%, respectively) [24]. Integrating WT1 PT-PCR and FCM methods has been shown to refine the predictive value of each technique [25,26]. Rossi et al. indicated that WT1 expression exhibits the optimal diagnostic performance when measured post-transplant, supporting its utility in predicting relapse at that juncture [21]. The lower specificity of post-SCT MRD by FCM compared to WT1 may be attributed to the presence of leukemia-associated immunophenotypes in regenerating bone marrow cells, reducing the sensitivity of aberrant phenotypes in identifying leukemic cells [27]. Our cohort confirmed the value of WT1 in disease monitoring, particularly noting its noteworthy negative predictive value for relapse.

The limitations of the study were that our cohort was of a medium size, displaying heterogeneity in transplantation dynamics and a relatively shorter follow-up duration. The advantage of our study lies in the execution of standard approaches within patients and treatment algorithms. The value of WT1 in MRD is increasingly being recognized, particularly for patients without unique fusion genes or mutations, offering potential guidance. The WT1 assay presents several advantages, including standardization, easy availability, limited operative bias, and subjective interpretation, with a sensitivity comparable to MFC in an allo-HSCT setting.

WT1 expression levels may serve as a valuable ancillary marker in MRD assessment and relapse prediction post-allo-SCT in AML patients, particularly for those lacking specific fusion genes or mutations. However, further large-scale, controlled studies are needed to standardize WT1 MRD assays and establish clear guidelines for their clinical application.

## Figures and Tables

**Table 1:** Demographics and clinical data of the patients.

Parameters	N = 58, %100
Age (median, min–max)	40 (17–62)
Gender (M/F)	34 (58.6%)/24 (41.4%)
WBC at diagnosis (10^9^/L) (median, min–max)	10.5 (0.69–361.9)
AML Type	
AML, NOS	41 (70.7%)
AML with recurrent abnormalities	8 (13.8%)
Therapy-related myeloid neoplasms	7 (12%)
Myeloid sarcoma	2 (3.4%)
Frequency of AML genetic abnormalities	
Isolated WT1 (+)	17 (29.3%)
FLT 3 (+)	21 (36.2%)
NPM1 (+)	9 (15.5%)
Inv 16 (+)	3 (5.2%)
t(8;21) +	4 (6.9%)
Karyotypic abnormalities	13 (22.4%)
Other cytogenetic abnormalities	5 (8.6%)
Cytogenetic risk group	
Favorable	1 (1.7%)
Intermediate	23 (39.7%)
Adverse	34 (58.6%)
Response to 1st induction	
CR	46 (79.3%)
REF	12 (20.7%)
Disease status at transplant	
CR1	42 (72.4%)
CR2	8 (13.8%)
Ref	8 (13.8%)
Donor type	
MRD (10/10–9/10)	35 (60.3%)
MUD (10/10–9/10)	21 (36.2%)
MMD (≥2)	2 (3.4%)
Donor gender incompatibility (male patient–female donor)	12 (20.7%)
Stem cell source (PBSC)	58 (100%)
HCT-CI comorbidity score (≥2)	4 (6.9%)
Conditioning regimen	
Busulfan based	32(55.2%)
Treosulfan based	23 (39.6%)
Others	3 (5.2%)
Conditioning regimen (MA/RIC)	48 (82.8%)/10 (17.2%)
CD34 (×10^6^ cell/kg)	6.96 (3.2–8.47)
Follow-up duration (months) (median, min–max)	21 (2–99)

Abbreviations: M: male, F: female, AML: acute myeloid leukemia, MA: myeloablative, RIC: reduced intensity conditioning, WBC: white blood count, NOS: not otherwise specified, CR: complete remission, REF: refractory; MRD: matched related donor, MUD: matched unrelated donor, MMD: mismatched donor, PBSC: peripheral blood stem cell, HCT-CI: hematopoietic cell transplantation-comorbidity index.

**Table 2:** WT1 measurement and disease course for patients’ remission.

Measurement Time (Median, Min–Max)	WT1 Assay (Copies/10^4^ ABL)	MFC	BME	Chimerism
At diagnosis	548 (4.5–9854)	49 (5–96)	70 (15–100)	N/A
Post-induction	11.6 (0–9356)	1.5 (0–75)	2 (0–90)	N/A
Pre-allo-SCT	7.91 (0–1453)	0.4 (0.01–75)	1 (0–90)	N/A
Post-allo-SCT 1st month	7.5 (0–721)	0.25 (0–4.5)	1 (0–6)	99.9 (90–99.9)
Post-allo-SCT 3rd month	8.41 (0–122)	0.27 (0–12)	1 (0–10)	99.1 (34.4–99.9)
Relapse	330 (22.9–2907)	20 (7–77)	30 (6–100)	90.7 (38.4–99.9)

N/A: not applicable, BME: bone marrow evaluation, MFC: multicolor flow cytometry.

**Table 3:** Correlation of WT1 and other parameters.

Parameters	Correlation Coefficient	*p*-Value
WT1 * MFC	0.578	<0.001
WT1high * MFC	0.537	<0.001
WT1 * BME	0.533	<0.001
WT1high * BME	0.565	<0.001
MFC * BME	0.758	<0.001
WT1 * Chimerism	−0.080	0.42
WT1high * Chimerism	−0.637	<0.001

BME: bone marrow evaluation, MFC: multicolor flow cytometry WT1: Wilms’ tumor gene 1. * it is association of both variables.

## Data Availability

The data are available upon request.
